# A home-based exercise intervention for caregivers of persons with dementia: study protocol for a randomised controlled trial

**DOI:** 10.1186/s13063-016-1582-z

**Published:** 2016-09-21

**Authors:** Wai Chi Chan, Nicola Lautenschlager, Briony Dow, Suk Ling Ma, Corine Sau Man Wong, Linda Chiu Wa Lam

**Affiliations:** 1 Department of Psychiatry, The University of Hong Kong, Queen Mary Hospital, Pokfulam, Hong Kong; 2 Academic Unit for Psychiatry of Old Age, Department of Psychiatry, University of Melbourne, Melbourne, VIC Australia; 3 NorthWestern Aged Mental Health, Royal Park Campus, Parkville, VIC Australia; 4 School of Clinical Neurosciences and the Western Australia Centre and Health and Ageing, University of Western Australia, Perth, WA Australia; 5 National Ageing Research Institute, The University of Melbourne, Melbourne, VIC Australia; 6 Department of Psychiatry, The Chinese University of Hong Kong, Tai Po Hospital, Tai Po, Hong Kong

**Keywords:** Home-based exercise, Tai Chi, Caregivers, Dementia, Depression, Randomised controlled trial

## Abstract

**Background:**

Family members, who provide the majority of care for persons with dementia, are especially vulnerable to developing depression. Interventions targeting their depressive symptoms have been proposed but their efficacies vary considerably. It has been suggested that interventions carried out in the home setting and involving both caregivers and care recipients are more efficacious. This study aims to compare the efficacy of a home-based structured exercise programme involving both persons with dementia and their caregivers with nonexercise social contact control in treating depression among caregivers.

**Methods/design:**

This is a parallel-group, assessor-blind, randomised controlled trial. A total of 136 caregiver-care-recipient dyads (i.e. 272 participants in total) will be recruited and randomly allocated to either a home-based structured exercise (sitting Tai Chi) group or a social contact control group. The trial comprises a 3-month intervention phase followed by an extended observation phase of another 3 months. All participants will be assessed at baseline, 6th week, 12th week and 24th week. The primary outcome will be the reduction in depression among caregivers as measured by the Hamilton Rating Scale for Depression. The secondary outcomes will be burden, quality of life, cognitive performance and balance ability of the caregivers, as well as the neuropsychiatric symptoms, cognitive function, balance and functional abilities of the persons with dementia. We will also examine whether the brain-derived neurotrophic factor gene modulates mood changes in response to exercise.

**Discussion:**

The findings offer a potential avenue of intervention by providing a low-cost, safe and effective treatment for depression among dementia caregivers, which may in turn also benefit the care recipients.

**Trial registration:**

ClinicalTrials.gov Identifier: NCT02132039, registered on 28 April 2014.

## Background

Dementia affects over 47 million people worldwide [1]. It causes decline in cognitive function, impairment in activities of daily living, and is associated with significant behavioural and psychological symptoms among the persons affected. Since the majority of dementia care is provided by family members [2], it is not surprising that they suffer from a high level of psychological distress and burden. It is now evident that informal caregivers of persons with dementia are at an elevated risk of developing depression and anxiety, and report poorer quality of life [3–5]. They also experience more stress than those who care for someone with physical disorders [6]. In a local study interviewing family caregivers of older adults with dementia in Hong Kong, they suffered significant stress and reported various negative emotions, conflicts between social roles, and difficulty in providing daily care and managing the care recipients’ behaviours [7]. Dementia caregiving has, therefore, emerged as a pressing health care issue.

In view of this, different interventions have been developed to alleviate depressive symptoms among caregivers of dementia persons. Recent reviews have analysed the efficacies of educational [8] and social support (e.g. befriending and peer support, family support and social network interventions, and support groups) [9], and psychological [10] and Internet-based interventions [11] for caregivers of persons with dementia. Though clinically meaningful, these interventions achieved only small effects [8, 11] or inconsistent results [9]. This may be partly because the programme goals and target groups were not clearly defined. Therefore, it has been suggested that research should focus on subgroups of caregivers such as depressed caregivers [12].

Among interventions for depression, exercise therapy has drawn much attention from researchers and practitioners. Exercise is a subset of physical activity that is planned, structured, repetitive and purposeful, and regular exercise compares favourably to antidepressants as treatment for milder depression [13]. Though its antidepressant mechanism is not yet clear, evidence suggests that exercise improves mood by normalising brain-derived neurotrophic factor levels [14], modifying serotonin function and releasing endogenous opioids [15]. More recently, exercise is reported to reduce atrial natriuretic peptide and brain natriuretic peptide, and to enhance copepetin and growth hormone among depressed individuals [16]. In addition, exercise improves one’s psychological and social sense of wellbeing [15]. It is, therefore, not surprising that a recent meta-analysis showed that exercise had a large and significant effect on depression (standardised mean deviation adjusted for publication bias = 1.11) with a fail-safe number of 1057 [17].

There has been emerging evidence supporting the effectiveness of exercise in treating depression in dementia caregivers. In a recent study, 17 caregivers participating in regular exercise of moderate intensity experienced less sense of burden and fatigue, and reported better sleep when compared with those in the control group [18]. Encouraging results have also been reported with endurance exercise [19], yoga [20], walking [21], and a combination of various physical activities [22].

However, the number of exercise studies for caregivers remains limited, and the sample sizes involved are usually small. This is probably related to the difficulty for dementia caregivers of participating in centre- or community-based exercise interventions because they are unable to find someone to take care of the care recipients, and lack time for additional activities [23]. In some previous studies, caregivers also voiced a preference for a programme that both caregiver and care recipient can participate in [22]. Home-based programmes involving both caregivers and care recipients may offer a possible solution [24]. Not only does the home-based dyadic intervention improve treatment adherence [25], it is also more successful in enhancing care-recipient function and reducing caregiver sense of burden [26].

We therefore propose to conduct the following assessor-blind randomised controlled trial of a home-based exercise intervention for both dementia caregivers and their care recipients.

### Objectives

The objectives of the study are as follows:

1. To evaluate the effect of a home-based exercise programme on:
  - caregivers’ depression
  - caregivers’ burden, quality of life, cognitive performance and balance ability
  - neuropsychiatric symptoms, cognitive function, and balance and functional abilities among persons with dementia
2. To assess whether the brain-derived neurotrophic factor (*BDNF*) gene modulates mood changes in response to exercise intervention

## Methods/design

### Design

**Fig. 1:**
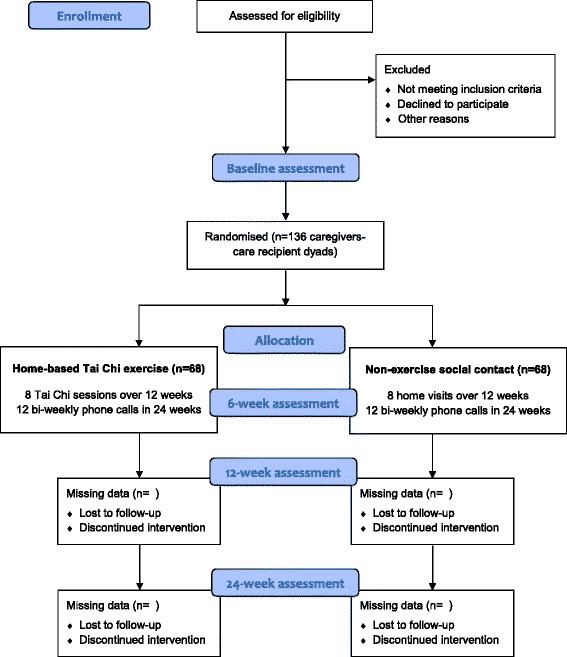
Consolidated Standards of Reporting Trials (CONSORT) flow diagram of the home-based exercise trial

This is a parallel-group, assessor-blind, randomised controlled trial, which comprises a 3-month intervention phase followed by an extended observation phase lasting for 3 months. Participants will be randomly assigned to either the home-based structured exercise (intervention) or the standard nonexercise social contact (control) group. The study is registered with ClinicalTrials.gov (NCT02132039), and conducted in accordance with the Declaration of Helsinki. Ethics approval has been sought from respective Institutional Review Boards. All results will be reported according to the Consolidated Standards of Reporting Trials (CONSORT) 2010 Statement [27]. A CONSORT flow diagram of the study is depicted in Fig. 1.

### Participants

Caregiver-care-recipient dyads will be recruited from psychogeriatric and geriatric outpatient clinics and various community centres for elders in Hong Kong by convenience sampling. Potential participants will be identified by the research team and screened for eligibility based on the inclusion and exclusion criteria. Informed consent will be obtained from all eligible participants before enrollment.

### Inclusion criteria

Caregivers:

- Aged 50 years or above
- Informal caregivers, i.e. unpaid family members who offer a substantial amount of care for the persons with dementia
- Geriatric Depression Scale (GDS) [28] score >0 but <8 (i.e. the local cut-off score for clinically significant depression)
- Understand Chinese
- Receiving antidepressant treatment on a steady dose for at least 3 months (if applicable)

Care recipients:

- Aged 60 years or above
- Dementia diagnosis confirmed by a physician
- Dependence in at least one of the activities of daily living (ADL)
- Mini Mental State Examination (MMSE) [29] score >10

### Exclusion criteria

Both caregivers and care recipients:

- No regular (i.e. at least three times/week) Tai Chi practice or other forms of mind-body exercise such as yoga, *qigong*, or mindfulness training in the past 6 months
- Presence of any condition which renders participants unsuitable for physical training, for example, severe psychotic symptoms, imminently suicidal, significant orthopaedic problems, or unstable medical conditions

### Randomisation and blinding

Participants will be randomly assigned to either the intervention or the control group using a computer-generated block randomisation sequence (block sizes of 4). Randomisation will be performed by an independent team member who will not be involved in the recruitment, assessment, or delivery of the intervention. The allocation sequence will be concealed from the study investigators and research staff in sequentially numbered, opaque, and sealed envelopes. The assessor for clinical parameters will be blinded to the randomisation status, and the trainer who conducts the intervention will be blinded to the assessment results.

### Intervention

The intervention consists of eight home-based sessions of sitting Tai Chi. Tai Chi is a traditional Chinese exercise that combines rhythmic and flowing patterns of bodily movements with coordinated breathing and mindful meditation [30]. It is a moderate-intensity aerobic exercise at about 3 to 6 metabolic equivalents [31]. It has been successfully implemented in the local older population [32] and, as a low impact exercise, it is associated with a lower risk of musculoskeletal injury making it a suitable form of exercise for older adults. Since the current study includes persons with significant cognitive impairment, we adopt sitting Tai Chi which is a simpler version of Tai Chi developed by Hong Kong Polytechnic University [33]. The exercise consists of 12 steps, which include weight shifting in different sitting positions, trunk and upper limb movements, and alternate thigh lift in a smooth and coordinated manner [33].

Four weekly exercise sessions will be arranged over the first 4 weeks, and four more biweekly sessions over the next 8 weeks. Each session will last for 1 h, which starts with 10 min of warm-up activities followed by 40 min of sitting Tai Chi practice and, lastly, 10 min of cool-down exercises. The caregiver-care-recipient dyads will practise Tai Chi in their home under the instruction of a Tai Chi teacher with more than 20 years of teaching experience, and a trained research assistant. In order to monitor the progress and address participants’ concerns, biweekly phone contacts will be made during the study period (12 scheduled phone contacts in 24 weeks). On completion of the eight home-based Tai Chi sessions, participants will be given a manual and training video with detailed instructions of the Tai Chi poses to facilitate their continuation of Tai Chi practices.

A standard nonexercise social contact will be provided to the participants in the control group. Since social contact may be beneficial to one’s mood and may contribute to any improvement observed in the intervention group, we provide participants in the control group with a level of social contact equivalent to the intervention group. They will be visited by the research assistant eight times over 12 weeks. The visits involve a series of conversations related to neutral topics that are designed according to the principles of a befriending programme [34]. These topics are structured and standardised, and do not involve any topic concerning physical activity. Similar to the intervention group, 12 biweekly phone calls will be made during the study period to offer a comparable level of social contact.

### Primary outcome

Participants will be assessed at baseline, 6th week, 12th week and 24th week for their responses to intervention. The assessment schedules for caregivers and care recipients are described in Table 1. The primary outcome of the study is the proportion of participants who are classified as responders by the Hamilton Rating Scale for Depression (HAM-D-17) [35]. HAM-D-17 is a widely used and reliable measure of depressive symptoms. The scores range from 0 to 52, with higher scores indicating greater depression severity. Response to the intervention is defined as a reduction of the HAM-D-17 total score by ≥50 % from baseline to endpoint. Assessors will follow the structured interview guide for the Hamilton Depression Rating Scale (SIGH-D) when administering the HAM-D-17 [36].

**Table 1:** Assessment schedule for caregivers and care recipients

	Baseline assessment	Week 6	Week 12	Week 24
Caregiver only				
GDS (screening only)	√			
HAM-D-17	√	√	√	√
ZBI	√	√	√	√
SF-12	√		√	√
EXIT25	√		√	√
IPIP	√			
Care recipients only				
CSDD	√	√	√	√
NPI	√		√	√
DAD	√		√	√
MBI	√		√	√
Caregivers and care recipients				
MMSE	√		√	√
Digit span	√		√	√
Delayed recall	√		√	√
CVFT	√		√	√
BBS	√		√	√
FR	√		√	√
TUG	√		√	√
Logbook	√	√	√	√
Pedometer	√	√	√	√
*BDNF* genotype	√			
Body weight and height	√		√	√
Blood pressure and pulse	√		√	√

*BBS* Berg Balance Scale, *BDNF* brain-derived neurotrophic factor, *CSDD* Cornell Scale for Depression in Dementia, *CVFT* Category Verbal Fluency Test, *DAD* Disability Assessment for Dementia, *EXIT25* Executive Interview, *FR* Functional Reach, *GDS* Geriatric Depression Scale, *HAM-D-17* Hamilton Rating Scale for Depression, *IPIP* International Personality Item Pool, *MBI* Modified Barthel Index, *MMSE* Mini Mental State Examination, *NPI* Neuropsychiatric Inventory, *TUG* Timed Up and Go, *ZBI* Zarit Burden Interview

Assessment for caregivers only:

1. The Zarit Burden Interview (ZBI) [37] is a 22-item self-administrated questionnaire specially designed to measure the stresses experienced by caregivers of persons with dementia. Caregivers will assess the impact of the patient’s disabilities on their life
2. The SF-12 Health Survey (SF-12) [38] measures the health-related quality of life and gives two component scores: a physical component summary (PCS) and a mental component summary (MCS)
3. The Executive Interview (EXIT25) [39] is a 25-item screening instrument developed to assess executive cognitive dysfunction
4. The International Personality Item Pool (IPIP) [40] consists of 50 items assessing the ‘Big Five’ personality traits: openness to experience, conscientiousness, extraversion, agreeableness, and neuroticism

Assessment for care recipients only:

1. The Cornell Scale for Depression in Dementia (CSDD) [41] is a clinician-rated instrument measuring depressive symptoms in persons with cognitive impairment
2. The Neuropsychiatric Inventory (NPI) [42] assesses the frequency and severity of neuropsychiatric symptoms across 12 domains including psychotic features, mood changes, activities disturbances and vegetative symptoms
3. The Disability Assessment for Dementia (DAD) [43] evaluates functional abilities in both basic and instrumental activities of daily living in people with dementia
4. The Modified Barthel Index (MBI) [44] measures individuals’ performance on 10 activities of daily living: eating, personal hygiene, bathing, toileting, dressing, bowel control, bladder control, transfers, walking, and stair climbing

Assessment for both caregivers and care recipients:

1. Sociodemographic information includes age, gender, education level and marital status
2. Lifestyle factors such as smoking, drinking, and sleeping habits. Health-related information like chronic health problems and medications will also be recorded. Resting blood pressure and heart rate will be taken. Body height and weight will also be measured to calculate Body Mass Index
3. Global cognitive function will be measured by the MMSE [29], digit span, delayed recall, and the Category Verbal Fluency Test (CVFT) [45]
4. Balance ability and functional mobility will be measured by the Berg Balance Scale (BBS) [46], the Functional Reach (FR) test [47], and the Timed Up and Go (TUG) test [48]
5. Physical activity and programme adherence will be assessed using logbook recordings. Before each assessment, participants will be asked to wear a pedometer for 1 week. Number of walking steps, history of falls, frequency, duration, and type of exercises during the week will be recorded in the logbook
6. Physiological markers – to examine the role of the *BDNF* Val66Met polymorphism in the antidepressant effect of exercise, buccal swabs will be collected at the baseline assessment

### Sample size calculation

The sample size is calculated by the proportion of caregivers who respond to the intervention as indicated by the HAM-D-17. Taking reference of the previous study [49], we expect a 30 % reduction in the proportion of participants rated as depressed in the control group with social contact only. Assuming that a further 30 % reduction is achieved by sitting Tai Chi, we need 56 caregiver-care-recipient dyads (i.e. 112 participants) in each arm to identify a significant effect at a two-tailed alpha of 0.05 and 80 % power. Allowing for 20 % dropout, the number of dyads in each arm will rise to 68. A total number of participants of 272 will be recruited.

### Statistical analyses

Baseline differences in demographic and clinical characteristics between the intervention and control groups will be evaluated by chi-square and independent sample *t* tests. All data will be analysed using the intent-to-treat principle. For outcome measures that are not normally distributed (e.g. the HAM-D-17), log transformation will be applied to perform the analyses. Mixed-effects models will be fitted for each outcome measure using baseline, 6th, 12th and 24th week follow-up data. Possible confounding factors such as sex, age, and use of antidepressant medication will be treated as time-dependent covariate in the mixed-effects models. If applicable, subgroup analysis will be carried out to estimate whether there is any gender difference (female versus male caregivers) regarding the effect of the intervention in alleviating depressive symptoms. Fixed effects for time, intervention group, and their interactions will be examined and unstructured covariance structure will be employed to account for the within-subject correlation over time. Levels of significance will be set at *p* < 0.05. All analyses will be performed using SPSS version 20.0 (SPSS Inc., Chicago, IL, USA).

## Discussion

Since the prevalence of dementia doubles every 5 years after the age of 60, population ageing inevitably increases the number of persons with dementia. Hong Kong is no exception to this trend. It is estimated that around one in 10 local older persons are now suffering from dementia [50].

Caregiving for persons with dementia is demanding and challenging. Evidence shows that it adversely affects caregivers’ physical [51] as well as mental wellbeing [3–5], including a vulnerability to developing depression. In a review of 10 studies involving 790 caregivers of persons with dementia, more than one fifth of them suffered from a depressive disorder [3]. The prevalence of depressive symptoms among caregivers is even higher [14].

This study will investigate the efficacy of a structured exercise programme in alleviating depressive symptoms among caregivers of persons with dementia. We focus on caregivers with depressive symptoms instead of depressive disorders as the former constitute the largest group of depressed caregivers. Besides, because of ethical concerns, we will refer those who suffer from clinically significant depression to appropriate agencies for psychiatric assessment and treatment.

The intervention is tailor-made for family caregivers in three ways. First, the programme is delivered at participants’ homes to facilitate their participation. Second, both caregivers and care recipients will be involved in the study. Third, a moderate-intensity aerobic exercise that has been successfully implemented in the local older population (i.e. sitting Tai Chi) is chosen as the intervention.

Sitting Tai Chi is a safe, low-impact and low-cost exercise intervention. If it is shown to improve caregivers’ mood, it will offer a potentially effective treatment alternative for depression among caregivers of persons with dementia. In addition, we expect that home-based exercise will also benefit the care recipients.

## Trial status

The study is still recruiting at the time of submission.

## Abbreviations

ADL: Activities of daily living
BBS: Berg Balance Scale
BDNF: brain-derived neurotrophic factor
CONSORT: Consolidated Standards of Reporting Trials
CSDD: Cornell Scale for Depression in Dementia
CVFT: Category Verbal Fluency Test
DAD: Disability Assessment for Dementia
EXIT25: Executive Interview
FR: Functional Reach
GDS: Geriatric Depression Scale
HAM-D-17: Hamilton Rating Scale for Depression
IPIP: International Personality Item Pool
MBI: Modified Barthel Index
MCS: Mental component summary
MMSE: Mini Mental State Examination
NPI: Neuropsychiatric Inventory
PCS: Physical component summary
REC: Research Ethics Committee
SF-12: SF-12 Health Survey
SIGH-D: Structured interview guide for the Hamilton Depression Rating Scale
TUG: Timed Up and Go
ZBI: Zarit Burden Interview
